# Oral and Subcutaneous Administration of a Near-Infrared Fluorescent Molecular Imaging Agent Detects Inflammation in a Mouse Model of Rheumatoid Arthritis

**DOI:** 10.1038/s41598-019-38548-0

**Published:** 2019-03-12

**Authors:** Sumit Bhatnagar, Eshita Khera, Jianshan Liao, Victoria Eniola, Yongjun Hu, David E. Smith, Greg M. Thurber

**Affiliations:** 1 Department of Chemical Engineering, University of Michigan, Ann Arbor, MI 48109 United States; 2 Department of Pharmaceutical Sciences, University of Michigan, Ann Arbor, MI 48109 United States; 3 Department of Biomedical Engineering, University of Michigan, Ann Arbor, MI 48109 United States

## Abstract

Rheumatoid arthritis (RA) is an inflammatory autoimmune disease that causes irreversible damage to the joints. However, effective drugs exist that can stop disease progression, leading to intense interest in early detection and treatment monitoring to improve patient outcomes. Imaging approaches have the potential for early detection, but current methods lack sensitivity and/or are time-consuming and expensive. We examined potential routes for self-administration of molecular imaging agents in the form of subcutaneous and oral delivery of an integrin binding near-infrared (NIR) fluorescent imaging agent in an animal model of RA with the long-term goal of increasing safety and patient compliance for screening. NIR imaging has relatively low cost, uses non-ionizing radiation, and provides minimally invasive spatial and molecular information. This proof-of-principle study shows significant uptake of an IRDye800CW agent in inflamed joints of a collagen antibody induced arthritis (CAIA) mouse model compared to healthy joints, irrespective of the method of administration. The imaging results were extrapolated to clinical depths *in silico* using a 3D COMSOL model of NIR fluorescence imaging in a human hand to examine imaging feasability. With target to background concentration ratios greater than 5.5, which are achieved in the mouse model, these probes have the potential to identify arthritic joints following oral delivery at clinically relevant depths.

## Introduction

Rheumatoid arthritis (RA), a form of inflammatory arthritis, is a chronic joint disease marked by pain and inflammation^1^ that affects 0.5% to 1% of the population worldwide^2^. Despite several existing treatments and recent advances in disease therapy, remission rates and morbidity remain a critical concern for RA patients^3^. Given the irreversible damage caused by joint inflammation and prevalence of effective disease modifying drugs, it is widely viewed that earlier treatment is needed for more effective management of RA. There is also some evidence that early intervention, specifically in cases of RA, has the potential for curative treatment^4^. However, current blood tests do not have adequate sensitivity and specificity for accurate diagnosis, and these tests do not provide insight into local joint conditions. Therefore, new quantitative diagnostic methods are needed to identify the pathology at an early stage to reduce disease morbidity^5^.

An inflamed synovial membrane is one of the earliest indications of the onset of RA^6^. Conventional imaging methods, such as ultrasonography, provide a valid assessment of synovitis^7^ but lack sensitivity for early arthritis^8^. Contrast enhanced magnetic resonance imaging (MRI), with its excellent soft tissue contrast, is able to provide details about inflammation within the joints and predict the disease progression at an early stage^9^, but this method is time-consuming and expensive for routine screening applications^10^. Optical molecular imaging is considered a promising alternative method for early stage RA detection. It has the advantage of providing specific molecular information without the use of ionizing radiation used in PET and SPECT molecular imaging methods. There have been several reported correlations between the severity of joint inflammation and fluorescence intensities from molecular probes^11–13^. Indocyanine green (ICG), a non-specific dye that binds to plasma proteins, has been studied in a few clinical reports since it is the only FDA-approved near-infrared (NIR) dye^14^. Although ICG has been reported to differentiate RA joints from healthy joints during later stages of the disease^15^, detection at the earliest stages, ideally subclinical inflammation for screening purposes, remains challenging and unrealized^12,16,17^.

To improve the feasibility of optical imaging for early detection of RA, a route of administration that is safe, inexpensive, and convenient for patients (to improve compliance) is ideal. Self-administration methods like subcutaneous injection (SC) and oral delivery (PO) are suitable for screening large portions of the population because they are generally safer than intravenous injections^18^, save time and medical personnel costs, and are preferred by patients^19,20^. Despite the advantages of alternative delivery routes, few studies have tested non-intravenous routes for administering molecular imaging agents (with topical application of PARP inhibitors^21^, now in clinical trials, being one of the few exceptions). This likely has to do with the challenges in designing agents with both efficient delivery and high specificity.

As a proof-of-principle study in mice to develop an imaging agent for self-administration and screening, we tested an integrin (α_v_β_3_) binding imaging agent that has been shown to efficiently target breast cancer in an orthotopic mouse model via oral administration^22^. We hypothesized that due to the high expression of α_v_β_3_ integrins on activated macrophages and the high macrophage infiltration in RA, we could use methods suitable for self-administration to detect inflammation of the joints in a collagen antibody induced arthritis (CAIA) mouse model of RA. These imaging agents are comprised of a targeting ligand that binds integrin of the form α_v_β_3_^23^ conjugated to a negatively charged NIR fluorophore. Given the different transport rates in the synovial tissue compared to tumors, we tested two methods of administration in mice: subcutaneous and oral delivery. To test the feasibility of imaging these targeted agents at clinically relevant depths, a three dimensional simulation (COMSOL Multiphysics) utilizing previously validated models of diffuse light fluorescence imaging^24,25^ was used to determine the necessary concentrations and targeting efficiency for early arthritis detection using epifluorescence imaging at clinical depths.

## Results

### Near-Infrared Integrin Imaging Agents Target Inflamed Joints after Subcutaneous Injection

**Figure 1:**
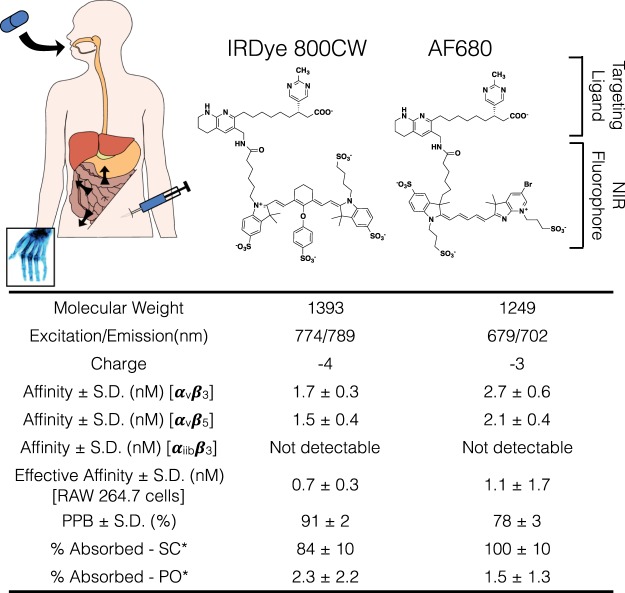
Routes of self-administration and physicochemical properties of the NIR imaging agents. *Percent absorbed for SC was measured by taking a ratio of the area under the curves of SC and IV delivery whereas the percent absorbed for PO was measured by urine collection due to the variable absorption of the imaging agents^22^. Plasma protein binding (PPB) data was published previously^22^.

Our previous work has demonstrated that the negative charge on imaging agents can facilitate targeting following oral delivery by increasing the target specificity, extending circulation in the body long enough to be taken up by antigen positive cells, retaining the probe in cells after receptor mediated internalization (due to their residualizing nature), and increasing oral absorption^22^. In this study, we employed this method for the detection of inflammatory arthritis in CAIA mice and studied the differences between two negatively charged imaging agents – IRDye800CW and AF680 agents – for more detailed structure-function relationships. The structure and physicochemical properties of the two imaging agents are shown in Fig. 1. The two agents have similar molecular weights, formal charge and binding affinities (Fig. S1), with the most notable difference being in their plasma protein binding (which can significantly impact distribution^26^).

**Figure 2:**
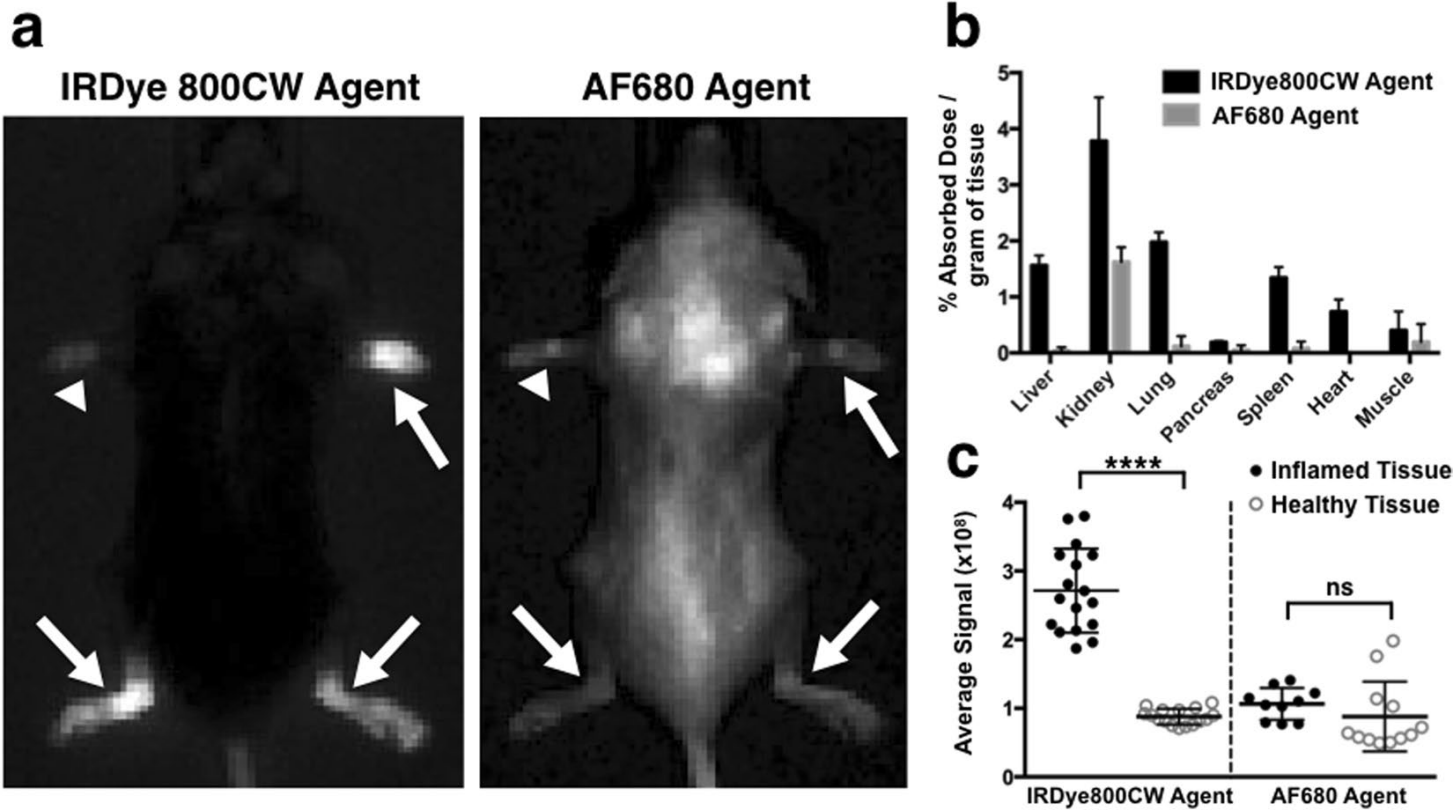
Subcutaneous Administration of IRDye800CW and AF680 Agents. (**a**) IVIS images taken at 48 hours post SC administration of the two agents with different fluorophores. Comparison of the (**b**) biodistribution and (**c**) average signals obtained from the inflamed and healthy paws and ankles of mice showed greater targeting of the IRDye800CW agent. ****Denotes p < 0.0001 and ns stands for not significant.

To study the effect of the physicochemical properties of the two agents on absorption and targeting, they were administered subcutaneously in a CAIA mouse model. Both agents are rapidly absorbed with 80% to 100% subcutaneous bioavailability. Images at 48 hours post SC injection (Fig. 2a) showed that the inflamed joints (arrow) had significantly higher signal than non-inflamed joint (arrowhead) for the IRDye800CW agent but were similar for the AF680 agent. Healthy mice were also injected with the same doses of both agents and showed significantly lower signal for the IRDye800CW agent (p < 0.0001) but no significant difference for the AF680 agent when compared to inflamed joints (Figs 2c and S2). The biodistribution data (Fig. 2b) shows lower uptake of the AF680 probe likely due to the faster clearance of the AF680 probe from the body (Fig. S3)^27^. The above results along with the higher oral absorption (Fig. 1) led to the selection of the IRDye800CW agent for oral delivery.

### Oral Delivery of IRDye800CW Integrin Agent Targets Macrophages in Inflamed Mouse Joints

**Figure 3:**
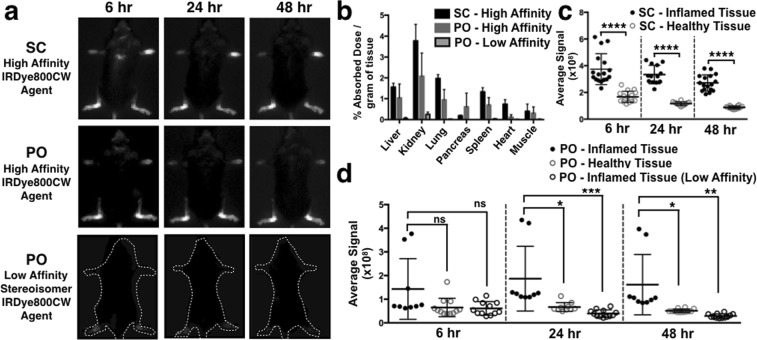
Oral versus Subcutaneous Administration of IRDye800CW Agent (**a**) IVIS images at 6, 24 and 48 hours of the IRDye800CW agent delivered subcutaneously (top), orally (middle), and the low affinity stereoisomer of the IRDye800CW agent delivered orally (bottom). Comparison of the (**b**) biodistribution and (**c**,**d**) average signals at varying times showing the specificity of the IRDye800CW agents in inflamed paws and compared to its low affinity stereoisomer. ****Denotes p < 0.0001, ***denotes p < 0.001, **denotes p < 0.01, *denotes p < 0.05 and ns stands for not significant.

The IRDye800CW agent showed high uptake in inflamed joints when delivered subcutaneously and orally (Fig. 3) in the CAIA mouse model. SC delivery gave the highest specific signal at 6 hours post administration versus 24 hours for PO delivery. This is likely attributed to the kinetics of absorption for the two routes of administration. The time of maximum plasma concentration for SC and PO delivery are 1 hour (Fig. S3) and 2.5 hours^22^ post administration, respectively. The signal in the healthy joints continued to drop with time, which increased the contrast between inflamed and healthy paws at 48 hours (Fig. S4). A low affinity stereoisomer of the targeting ligand was conjugated with IRDye800CW and dosed orally in mice with inflamed joints. The low uptake of low affinity agent in the joints of these mice confirmed the targeting specificity of the high affinity agent. The biodistribution data was similar for the high affinity agent irrespective of the route of delivery and significantly lower for the low affinity binder.

**Figure 4:**
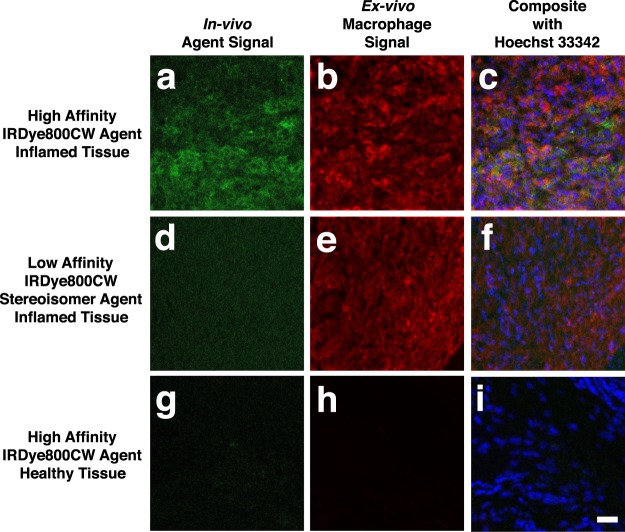
Confocal microscopy images of (**a**) high affinity IRDye800CW agent uptake corresponding with (**b**) macrophage presence in inflamed ankles. Less contrast was apparent with the low affinity IRDye800CW stereoisomer uptake (**d**) in macrophages in inflamed ankles (**e**). Healthy mice administered the IRDye800CW agent show (**g**) low signal in healthy ankles and (**h**) a lack of macrophage staining. Composite images of the IRDye800CW agent, macrophages and Hoechst (blue) showing the presence of cells (**c**,**f**,**i**). Autofluorescence controls are shown in Fig. S5. Scale bar = 20 μm.

*Ex-vivo* labeling of histology slides showed macrophage infiltration in the inflamed ankles and lack of staining in the healthy ankles of control mice (Fig. 4). The presence of macrophages correlated with significant uptake of the orally delivered IRDye800CW agent in inflamed ankles (Fig. 4a). Low uptake of the low affinity stereoisomer in inflamed ankles and low uptake of the high affinity agent in healthy (control) ankles highlighted the specificity of the imaging agent (Fig. 4d). The signal intensities for all images were significantly higher than autofluorescence controls (Fig. S5).

### Computational Modeling Indicates Mouse Model Targeting Efficiencies are Sufficient for Detection when Scaled to Clinical Depths

**Figure 5:**
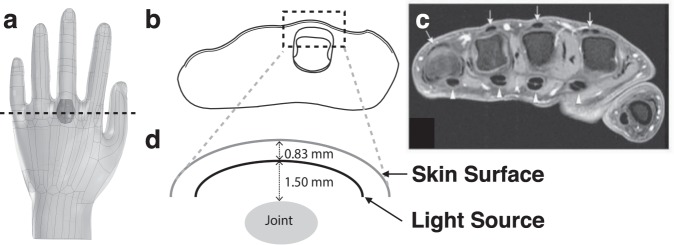
Schematics of hand and joint placement used in the COMSOL model. (**a**) CAD file of the hand and synovial space with 0% swelling. (**b**) A cross-sectional view (at the dotted line in A) of the synovial space. (**c**) MRI of the metacarpophalangeal joints from Nieuwenhuis *et al*.^52^. (**d**) Schematic of the dotted box in B showing the relative distances of the light source from the skin surface and the joint.

A potential limitation of optical imaging for detecting clinical RA is the depth of imaging of NIR fluorescence using epifluorescence detection (compared to tomographic reconstruction). To facilitate translation between the animal model results and clinical depths, a CAD model of a human hand was used to simulate NIR epifluorescence imaging of targeted molecular imaging agents in the synovial tissue at clinically relevant depths (Fig. 5a). Based on the thickness of the tissue relative to the scattering length of NIR light, the diffusion approximation was used along with a light source located one scattering length within the top surface of the hand (Fig. 5b,d). The joint location is below this depth based on MRI images (Fig. 5c). High resolution images of the hand joint^28^ were used to generate a 3D model of the synovial space where molecular imaging agents would bind their target during inflammation. The shape and thickness of the synovial space was based on sagittal, coronal and transverse images of the joint (Fig. S6).

**Figure 6:**
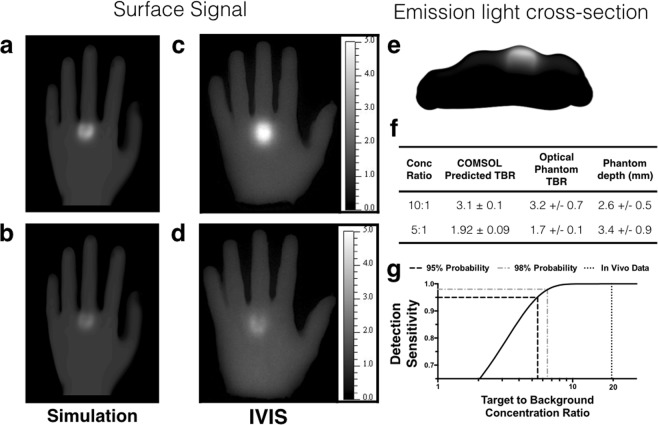
Simulation results from the COMSOL model and fluorescent optical phantom images. COMSOL simulation results with a concentration ratio of (**a**) 10:1 and (**b**) 5:1 between the synovium and the rest of the tissue. Optical phantoms imaged on an IVIS with concentration ratios of (**c**) 10:1 and (**d**) 5:1. (**e**) A cross-sectional view of the emission profile following epifluorescence excitation from the top surface in a COMSOL simulation with a 10:1 concentration ratio between the synovium and surrounding tissue. (**f**) A comparison of the COMSOL predicted TBR and optical phantom TBR. (**g**) Required concentration ratio for positive identification based on the z-statistic and the estimated concentration ratio achieved in the mouse model.

A two-step simulation in COMSOL was employed where the excitation light was first simulated from a planar source shining on top of the hand and used to calculate the fluorescent dye absorption and emission. The excited fluorophore then acted as the source for the emission light simulation. The fluorescent dye concentration in the synovial tissue was varied while the concentration in the remainder of the tissue was kept at a constant level to capture background autofluorescence and non-specific probe accumulation in healthy tissue. Surface fluence was used to determine the image intensity while varying the concentration ratio between the synovial tissue and hand. Optical phantoms (Fig. S7) were used to validate the simulations, and the experimentally measured target to background ratios (TBR) were similar to those predicted by the simulation (Fig. 6). The concentration ratios between the synovial space and surrounding tissue (e.g. 10:1) were proportional to the TBR as expected (Fig. 6f). The discrepancy between the optical phantom and the simulations likely resulted from the increased experimental depth of the joint (Fig. 6f) compared to the depth of 2.3 mm in the simulation.

For positive identification of an inflamed joint, the signal contrast between the joint space and background tissue must be higher than the background variability (the contrast to noise ratio). To obtain a reasonable estimate for the background variability, reported mouse and clinical data were used to estimate the standard deviation in background signal. These data were taken at long times after administration (24 hours post-injection) to minimize variability due to transient redistribution from the blood. Assuming that the variability correlates with the background signal (i.e. a higher absolute background signal results in higher absolute variability such that the standard deviation is proportional to the signal), a previous report in mice indicated a background variability of 64% for antibodies 72 hours after delivery^25^. A clinical imaging sample indicated a background variability (after subtracting non-tissue signal from the *in vivo* background signal and error propagation) of 51% at 24 hours post-injection of the small molecule ICG^29^. While the actual values may vary due to the reasons listed above, 64% variability in the background intensity was chosen as a reasonable estimate of late imaging time background standard deviation. This is a conservative estimate, since the variability in the healthy paws at 48 hrs after administration for the current data was only 12.5% (Fig. 3). Since the average signal from the joint would be compared to a background signal with a measured standard deviation, the threshold contrast to noise ratio (CNR) for detection was calculated using a z statistic. From the cumulative normal distribution table, a CNR of 1.65 gives a 95% probability of positive signal relative to the background. Therefore, interpolating the CNR values from Table S3, probes with 5.5:1 concentration ratios between synovial tissue and background tissue are necessary for positive detection of inflammation (Fig. 6g). Based on the estimated concentration ratios from mice (Fig. S8), this level of contrast is achieved with these agents following both PO and SC delivery.

## Discussion

Early detection of RA could enable therapeutic intervention before the disease has caused significant irreversible damage, thereby improving patient outcomes. Clinical data suggest that it may even be possible to cure the disease if diagnosed early and accurately^30^. Blood markers lack the sensitivity and specificity necessary for early diagnosis, and clinical scores cannot provide quantitative precision or detect subclinical inflammation. Therefore, researchers are exploring a variety of imaging agents including ^99m^Tc-MDP^31^, PSVue 794^32^, αCD11b-APC combined with MMP750^33^, NIR2-folate^34^, MMP-3 specific polymeric probe using NIRF^35^, protease activated NIRF probes (sensitive to Cathepsin B)^11^, integrins^36,37^ and AP39-TSC^38^. Research into new imaging agents continues in order to develop probes capable of detecting this early stage of the disease.

NIR fluorescence molecular imaging has the potential to detect joint inflammation in a minimally invasive manner by providing both spatial and quantitative molecular information within the joints. This technique has several advantages over ultrasound by simultaneously capturing data from multiple joints using FDA approved NIR hand scanners and yielding quantitative molecular information from targeted probes. It is also less time consuming, less expensive to use and employs non-ionizing radiation compared to other frequently used imaging modalities like MRI, PET, and SPECT. Ultimately, the accuracy and advantages will have to be tested in a clinical setting. Current clinical efforts for optical imaging have focused on non-targeted ICG given its FDA approval. However, the low contrast provided by this non-specific protein-binding dye requires dynamic imaging^39^ to parse increased uptake in the joints from normal background and surface vessel fluorescence. Targeted imaging could provide significantly higher contrast ratios for more definitive image analysis, diagnosis, and follow up testing. However, many of these methods include intravenous delivery and/or radiolabels, and the required imaging agent uptake ratios and molecular targets have not been analyzed in a quantitative fashion over clinically relevant length scales. In this work, we used an integrin binding ligand conjugated with a NIR fluorophore and tested its ability to detect RA in a CAIA mouse model using SC and PO delivery. We also simulated NIR fluorescence imaging of targeted synovial tissue in a human hand while varying the target to background ratios using COMSOL Multiphysics (Table S3) to determine the optical feasibility of translating this technique to humans.

Oral and subcutaneous delivery, when used for screening applications, have several potential advantages over intravenous delivery of imaging agents, including safety, cost, and compliance. Intravenous delivery generally carries a higher risk of adverse reactions^18,40^, and while the absolute values are low, the risk in screening large numbers of patients in a healthy population requires a large safety margin. Additionally, even the fastest distributing affinity ligands require imaging to take place several hours post administration to allow for the imaging agent to clear from the body, which results in multiple or extended visits to the clinic. These visits can lead to increased costs owing to the health care professional time required to dose the patients in a sterile manner and monitor them in case of adverse events. Self-administration of these imaging agents would allow for a single visit to the clinic, outside of the specialized and infrastructure-heavy hospitals^41^ for imaging, thereby mitigating the costs of screening. Currently, there is a lack of clinical data using alternative routes of administration for imaging agents, and more investigation is warranted to see if these advantages are realized in a clinical setting.

Due to their efficient targeting and optical properties, the IRDye800CW and AF680 agents were delivered by SC and PO administration in a CAIA mouse model. We tested the affinity of these agents to three different human integrin heterodimers α_v_β_3_, α_v_β_5_ and α_iib_β_3_, in a plate assay to provide insight into the likelihood of these agents binding to human integrins in patients in a clinical setting. Although the targeting ligand has high specificity for α_v_β_3_ over α_v_β_5_^23^, the modification with these two dyes increased the cross-reactivity with α_v_β_5_, which is also upregulated on macrophages^42^. The high affinity of the IRDye agent to mouse α_v_β_3_ on macrophage cells (RAW 264.7 cells) and slower plasma clearance are consistent with the higher uptake of the agent by activated macrophages in RA when compared to the AF680 agent. Interestingly, the affinity of the AF680 probe was much higher for human α_v_β_3_ in the context of cells compared to the plate assay (Fig. S1). No binding to α_iib_β_3_ was detected (Fig. 1), which is an important safety aspect to avoid binding to platelets^43^. Both the agents are structurally very similar, with the AF680 agents having the benefit of faster clearance from the body, which reduces background signals. However, the IRDye800CW agent performed significantly better than the AF680 during *in vivo* experiments, which could be due to several reasons. Rapid clearance is less beneficial in applications when there is a significant absorption phase, such as SC or PO delivery, in contrast to IV delivery^27^. The clearance during the absorption phase results in lower maximum concentrations within the body leading to less uptake of the agent in the target tissue. The autofluorescence in the 680 channel is also higher than the 800 nm channel, compounding the issue of low concentrations in the blood.

The IRDye800CW agent showed specific targeting in the CAIA mouse model relative to healthy controls when delivered subcutaneously, and given the similar kinetics following oral delivery, we hypothesized that it would provide specific targeting following PO administration. Figure 3 confirmed this hypothesis with the major difference between the two being the higher variability in target tissue and healthy control tissue following oral delivery. The low-affinity stereoisomer of the IRDye agent had lower uptake in inflamed tissue compared to the high affinity probe uptake in healthy mice, indicating some binding of the high affinity agent to integrins in the healthy tissue. Microscopically, significant uptake is seen in macrophages in inflamed joints (Fig. 4).

Both delivery methods produce signals higher than seen in healthy control joints, especially at later times. The subcutaneous route results in less variability in absorption; however, both the signal and background intensities are proportional to the absorbed dose, so the TBR is expected to be similar between both routes. Two (out of 11) mice had low plasma concentrations (<2 nM) 6 hrs post administration at the 5 mg/kg dose with insufficient signal for detection in the inflamed joints (Fig. S9). The lack of sufficient absorption (0.6 and 1.2 nM concentrations versus > 2 nM for mice with sufficient signal) was not seen at this dose level in nude mice with orthotopic xenografts (n = 6)^22^. However, it is unclear if this is due to differences in the mouse strain or a statistical anomaly. Since background signals linearly change with the absorbed dose, measuring the signal in background tissue (e.g. muscle) would be a facile way to determine if there was sufficient absorption of the agent. In cases where the background signal is below a threshold that identifies sufficient absorption, the oral dose could be increased or the imaging agent could be given subcutaneously. Additionally, the formulation of these agents (currently in water) could be optimized to decrease variability.

Based on the targeting efficiency achieved in the animal experiments, we used computational modeling to scale the results to clinical depths. The ability to detect the fluorescence signal over this length scale is a necessary, albeit not sufficient, criterion to scale to the clinic. This was achieved using an optical imaging COMSOL model to simulate various target to background concentration ratios and different extents of joint swelling (Fig. 5 and Table S3). Diffuse light simulations are well-established in the field of biophysics^44^, and similar models have been used for whole animal NIR tomography^45^ to test the depth of detection in NIR fluorescence tomography of breast tissue^46^ and epifluorescence imaging of ICG in arthritis^12^. The results showed that the TBR and CNR values are strongly affected by the target to background concentration ratio and only weakly impacted by the extent of joint swelling (i.e. thickness of the synovium) (Table S3). The regions of interest used to calculate the TBR and CNR avoided the diffuse light at the edges of the joint. The optical phantom experiments were conducted to validate the *in silico* results with excellent agreement (Fig. 6). The variability in the optical phantom values was likely due to differences in the depth of the synovial tissue in the physical model. For positive identification of inflammation, the probability of identification is based on the contrast to noise ratio (CNR). Since the location of the region of interest (the joint) are known a priori (in contrast to cancer screening), a CNR of 1.65 is needed for 95% confidence in the detection of joint inflammation. This corresponds to a target to background concentration ratio of ~5.5:1. By scaling the concentrations from the CAIA mouse model, the experimental values in the mouse model were ~4 times larger than the theoretical requirement and should be sufficient for detection at clinical depths provided the targeting efficiency is similar.

While these proof-of-principle studies are promising, the model and approach have several limitations and areas for improvement. The most notable are the requirement to scale these results to the clinic and to differentiate rheumatoid arthritis from other inflammatory arthritis conditions. The animal experiments in this paper show that integrin binding imaging agents can detect inflammatory arthritis after oral and SC delivery in a mouse model of RA and to our knowledge, are only the second report of oral NIR imaging agent delivery. However, the results need to be scaled up to larger animal models and eventually the clinic to demonstrate acceptable absorption and targeting in larger species. Clinical data showing efficient imaging of radiolabeled integrin targeting agents in RA (e.g.^37,47^) provide some support, but the kinetics will have to be analyzed after oral delivery.

When translating this technique to humans, an important clinical consideration is distinguishing other causes of joint inflammation, such as ankylosing spondylitis. Although the clinical presentation of these diseases is distinct, differential diagnosis at an early stage can be challenging. Clinical data from RGD-based integrin imaging agents shows higher uptake in RA than osteoarthritis patients^37^. However, these agents will likely bind activated macrophages in other inflammatory arthritic conditions. If this occurs, it could be circumvented by several approaches, which are important future steps to improve the robustness of the imaging results and leverage the full capabilities of molecular imaging. First, it may be possible that a more quantitative and objective measure of inflammation from imaging will be able to better differentiate these conditions compared to a clinical exam alone. Second, recent results have demonstrated that dyes in the ~750–900 nm wavelength range (often referred as NIR window I) can be imaged in the 1100–1400 nm emission range (the NIR II window)^48^. The lower scattering results in higher resolution of structures right under the skin. Higher resolution may enable additional critieria (e.g. tenosynovitis^49^) to be explored for diagnosing the cause of inflammatory arthritis. Although ultrasound (US) is more time-consuming than NIR fluorescence imaging (which takes only seconds to image all the joints of both hands), the similar (low) cost^50^ and lack of ionizing radiation (plus strong clinical precedent) make this an attractive modality to pair with NIR imaging. Similar to PET/CT, NIR/US imaging would combine the molecular imaging data from NIR fluorescence with the anatomical and physiological information from US. As an alternative to the above methods of analyzing the spatial location and intensity of inflammation in the joints and surrounding tissue, pairing the current agent, which targets a marker of inflammation for high sensitivity, with a second NIR agent that binds a different target for improved specificity could increase the overall diagnostic ability of the technique. It is important to keep in mind that all these criteria would need to be measured through a controlled clinical trial to determine if they can accurately distinguish RA for inflammatory arthritis at an early stage.

In conclusion, oral and subcutaneously delivered NIR fluorescence molecular imaging agents (routes that enable self-administration) are able to detect joint inflammation in a CAIA mouse model at contrast levels sufficient for imaging at clinical depths. Subcutaneous delivery shows less variability in absorption, while contrast levels between SC and PO delivery are similar. These delivery routes have the potential for improved safety, low cost, and higher compliance due to ease of use compared to intravenous injection and warrant further study.

## Methods

### Synthesis

Imaging agents were synthesized as previously reported^22,27^. Briefly, the targeting ligand ester (ChemPartner; Waltham, MA) was hydrolyzed to a carboxylic acid with 150 μL of ethanol and 7 μL of 1 M NaOH per mg of the drug overnight. The resulting mixture was neutralized, evaporated, re-dissolved in DMSO, and reacted with the NHS ester form of IRDye 800CW (LI-COR; Lincoln, NE) and Alexa Fluor 680 (Life Technologies; Carlsbad, CA) in a 2:1 molar ratio in the presence of TEA (Sigma Aldrich; St. Louis, MO). The reaction was run overnight and purified using a preparative scale Luna C18(2) column (Phenomenex; Torrance, CA) on a Shimadzu reverse phase HPLC. The purified product was then run on MALDI to confirm the presence of the product^22^.

### Cell Lines

MDA-MB-231 cells and RAW 264.7 cells were obtained from ATCC (Manassas; VA) and grown in DMEM with 10% FBS (Life Technologies; Carlsbad, CA) and 1% Penicillin-Streptomycin (Life Technologies; Carlsbad, CA).

### Imaging Agent Properties

Integrins (human α_v_β_3_, α_v_β_5_ and α_IIb_β_3_) were obtained from R&D Systems (Minneapolis, MN). These were resuspended in PBS at concentrations recommended by the manufacturer. The integrins were diluted in PBS at a concentration of 4 μg/ml and 100 μL was pipetted into a 96 well plate protein capture plate (Thermo Fisher Scientific; Waltham, MA; Cat. No. 3855) and placed at 4 °C overnight. The wells were washed three times with PBS-Tween (0.05%) and then incubated with PBS-BSA (2%)-Tween (0.05%) for 2 hours at room temperature on an orbital shaker. The wells were then washed three times with PBS-Tween (0.05%) and incubated in the imaging agent at varying concentrations in PBS-BSA (1%)-Tween (0.05%) for 1 hour at room temperature on the orbital shaker. The wells were washed with PBS-Tween (0.05%) and imaged on the Odyssey CLx (LI-COR; Lincoln, NE).

The binding affinity on cells was measured as previously reported^22^. The cells were harvested and incubated with varying concentrations of the agents in cell culture media on ice for 3 hours. The cells were then washed with 0.1% PBS-BSA and run on an Attune acoustic focusing cytometer to quantify the fluorescence. The equilibrium dissociation constant (binding affinity) for both assay types was obtained by fitting the data on Prism (GraphPad Software; La Jolla, CA).

### *In Vivo* Experiments

All animal experiments were approved by the University of Michigan Insitutional Animal Care and Use Committee (IACUC) and conducted in accordance with guidelines from the National Institutes of Health, US. To determine the absorption following SC administration, 1.5 nmoles of the imaging agent (~0.1 mg/kg) were administered in 8–10 week old female C57BL/6 J mice via SC and intravenous (IV) injection (n = 3 for each) (Fig. S2). Retro-orbital blood draws were conducted at 5, 15, 30, 60 120, 240, 360 and 1440 minutes post-administration for SC delivery and at 1, 3, 5, 15, 30, 60, 180, 360 and 1440 minutes post-administration for IV delivery. A 10-μl aliquot of blood was mixed with 20 μl PBS-EDTA and spun down at 2000 g for 1 minute. The plasma (supernatant) was then pipetted into a 384 well plate and the fluorescence of the sample was measured on the Odyssey CLx. The signal was converted into a plasma concentration of the imaging agent using a calibration curve in mouse plasma. Area under the plasma concentration-time curves (AUCs) for the mice were calculated using the trapezoidal rule and the bioavailability was determined by the ratio of the AUC of SC to IV. For PO delivery, the bioavailability was measured using a metabolic cage as previously reported in Bhatnagar *et al*.^22^ (n = 3).

The collagen antibody induced arthritis (CAIA) model was used for imaging RA in mice (Chondrex; Redmond, WA). 1.5 mg of the antibody cocktail was administered by intraperitoneal (IP) injection into 8–10 week old female Balb/c mice (Jackson Laboratories; Bar Harbor, ME). Three days later, 50 μg of LPS was administered via IP injection. The extent of paw and ankle inflammation was measured using calipers, and once inflammation was detected (8–10 days after the antibody injection using caliper measurements of the paw and ankle), the mice were administered doses of the imaging agents.

Imaging agents were administered by either SC injection (dorsal side between the shoulder blades) or PO to the CAIA model mice with inflamed joints (n = 3 for each delivery route) or control mice (n = 3 for each delivery route) with no inflammation. For SC injections, the mice were anesthetized using 2.5% isoflurane and the imaging agents were either co-administered at a dose of 1.5 nmole each in the same mouse or dosed individually at 1.5 nmole. For PO, mice were fasted for 6 hours pre-administration. A 24-gauge feeding needle (Fine Science Tools; Forster City, CA) was used to administer a 75 nmole dose. Post injection, the mice were imaged on an IVIS Spectrum (Perkin Elmer; Waltham, MA) at 6, 24 and 48 hours. After the 48-hour image, the mice were euthanized and all organs were resected for measuring biodistribution. The ankle was snap frozen in OCT using isopentane cooled on dry ice for histology. The biodistribution protocol is previously reported^22,51^, but briefly the organs were minced with a razor blade, weighed, and digested in Eppendorf tubes using a collagenase (Worthington Biochemical; Lakewood, NJ; Cat. No. CLS-4) solution (5 mg/ml) in RIPA buffer (Boston BioProducts; Ashland, MA) at 37 °C for 20 minutes. The tissue was sonicated, digested for 30 minutes at 37 °C using a 50:50 trypsin and RIPA buffer solution, and sonicated again. A dilution series of the resulting mixture was scanned in a black walled 96-well plate using an Odyssey CLx. Absolute quantification was obtained by comparison with a calibration curve. The uptake values were normalized to the average amount of probe that reached the systemic circulation.

### Statistical Methods

The data shown in Figs 2c and 3d was obtained by measuring the intensity of the imaging agent in paws and ankles that had measurable inflammation (>0.1 mm) and comparing it to the intensity in the paws and ankles of healthy control mice that were dosed with the imaging agent. The data (n = 3 mice for each cohort) was then analyzed on Prism (GraphPad Software;La Jolla, CA) using an unpaired t-test to obtain p values.

### Histology

The frozen ankles were sectioned into 10 μm slices on a cryostat using a tungsten carbide blade. The tissue was not fixed and decalcified to avoid loss of fluorescence signal. The slices were stained *ex-vivo* with an anti-Mac3 antibody (BD Biosciences; San Jose, CA; Cat. No. 553322) labeled with Alexa Fluor 680 (Life Technologies; Carlsbad, CA) and Hoechst 33342 (Thermo Fisher Scientific; Cat. No. H3570). The slides were then washed with PBS and imaged on an Olympus FV1200 confocal microscope with 405, 633 and 750 nm laser lines.

### Governing Equations for COMSOL Model

The computational model of near-infrared fluorescence uses a steady state diffusion equation to approximate the propagation of light in highly scattering media based on the thickness of the human hand and high albedo of NIR light^25,28,52^:

$$\nabla \cdot -\,D\nabla u+{\mu }_{a}u=f$$

where *u* is the fluence rate of light, *D* is the diffusion coefficient, ${\mu }_{a}$ is the absorption coefficient, and *f* is the light source. Parameters used for the simulation are listed in Tables S1 and S2. The problem is divided into two simulation steps: excitation followed by emission. For the excitation light:

$$\nabla \cdot -\,D\nabla u+{\mu }_{a}u=0$$

The planar light source is positioned at one scattering distance below the top surface of the hand, where the scattering of the light is assumed to be isotropic (e.g.^25,53,54^). Fluorophores in the hand are excited by light based on their absorption coefficient and the photon density of the excitation light. They emit fluorescence signals based on their quantum yield, which propagates through the tissue and can be detected at the surface of the hand. Autofluorescence in the hand is also included in the model as an ‘equivalent dye’ concentration in the tissue. For the emission light:

$$\nabla \cdot -\,D\nabla u+{\mu }_{a}u=\eta \cdot {\mu }_{fx}\cdot u\cdot {[C]}_{{joint}}$$

Both the excitation and emission light simulations use Robin boundary conditions at the outer surface of the hand.

$$-{\rm{n}}\cdot -\,D\nabla u-u/(2\cdot Cr)=0$$

### CAD Drawing of Hand MCP Joint Using Solid Works

To obtain results at clinical relevant depths, a CAD hand file (GrabCAD; Cambridge, MA) was used and scaled to the size of a human hand. The planar light surface was created by duplicating the top surface of the hand using SolidWorks (SolidWorks; Concord, MA) and translating that surface one scattering distance beneath the top of the hand (Fig. 5a–f). Next, the synovial space around the MCP joint in the middle finger of the hand was made using SolidWorks. The size and shape of the membrane (Fig. S6a–c) was based on sagittal, coronal and transverse MR images of the MCP joint (Fig. S6d–f). The size of the base case synovial membrane is referenced from the scale bar on the coronal and transverse views of the MR images. The membrane position in the hand is determined from the MR image (Fig. 5a,d).

### Development of COMSOL Model

The Helmholtz equation in COMSOL Multiphysics (COMSOL Inc.; Burlington, MA) was used for simulating the light diffusion equation at steady state. The constructed CAD model was imported into COMSOL, and the parameters and boundary conditions of the diffusion equation were correspondingly assigned. The simulation involves two Helmholtz equations depicting the excitation and emission light separately. The finest tetrahedral mesh was used, and COMSOL solved the differential equations at a tolerance of 10^−5^ for four iterations. The simulated results were saved as images under volume mode for image post-processing.

### Imaging Optical Phantoms

An optical phantom was used to validate the COMSOL simulations (Fig. 6). The same CAD file used for the COMSOL simulations was also used to make a replica of the synovial space using 3D printing (Cube 2, Cubify). A silicone mold of a hand and the 3D printed synovial space (Fig. S7) was used to generate optical phantoms, which consisted of 3% agarose gel (for structure), 1% Intralipid (Baxter, Deerfield, IL) and 50 ppm India ink to mimic the absorption and scattering properties of human tissue^25^. IRDye 800CW carboxylic acid (LI-COR Biosciences; Lincoln, NE) was added at a concentration of 10 nM to the hand to represent background signal and 50 nM or 100 nM to the synovial cavity to represent specific uptake. The synovial cavity was placed ~2.5 mm from the surface of the hand. Once the gel solidified, it was imaged on an IVIS Spectrum (Perkin Elmer; Waltham, MA) and the TBR was calculated using the LivingImage software (Perkin Elmer; Waltham, MA).

## Supplementary information

Supplementary Info

## Data Availability

All data generated or analysed during this study are included in this published article (and its Supplementary Information files).
