# Supplementary material for: Oral and Subcutaneous Administration of a Near-Infrared Fluorescent Molecular Imaging Agent Detects Inflammation in a Mouse Model of Rheumatoid Arthritis
Source: Sci Rep. 2019 Mar 12;9:4661. doi: 10.1038/s41598-019-38548-0 (PMC6411963; doi:10.1038/s41598-019-38548-0)
Supplement: Supplementary file 1 — Supplementary Info [file 41598_2019_38548_MOESM1_ESM.pdf]

Supporting Information:

Oral and Subcutaneous Administration of a Near-Infrared Fluorescent Molecular Imaging Agent Detects Inflammation in a Mouse Model of Rheumatoid Arthritis

Authors: Sumit Bhatnagar[1,#], Eshita Khera[1,#], Jianshan Liao[1,§], Victoria Eniola[1,w], Yongjun Hu[2], David E. Smith[2], and Greg M. Thurber[1,3,*]

[#]Equal Contribution

Affiliations:

[1] Department of Chemical Engineering, University of Michigan, Ann Arbor, MI 48109.

[2] Department of Pharmaceutical Sciences, University of Michigan, Ann Arbor, MI 48109.

[3] Department of Biomedical Engineering, University of Michigan, Ann Arbor, MI 48109.

* Corresponding author:

Greg M. Thurber
Email: gthurber@umich.edu
2800 Plymouth Rd.
Ann Arbor, MI 48109
T: 734-764-8722

Figure S1. Binding Affinity of Imaging Agents

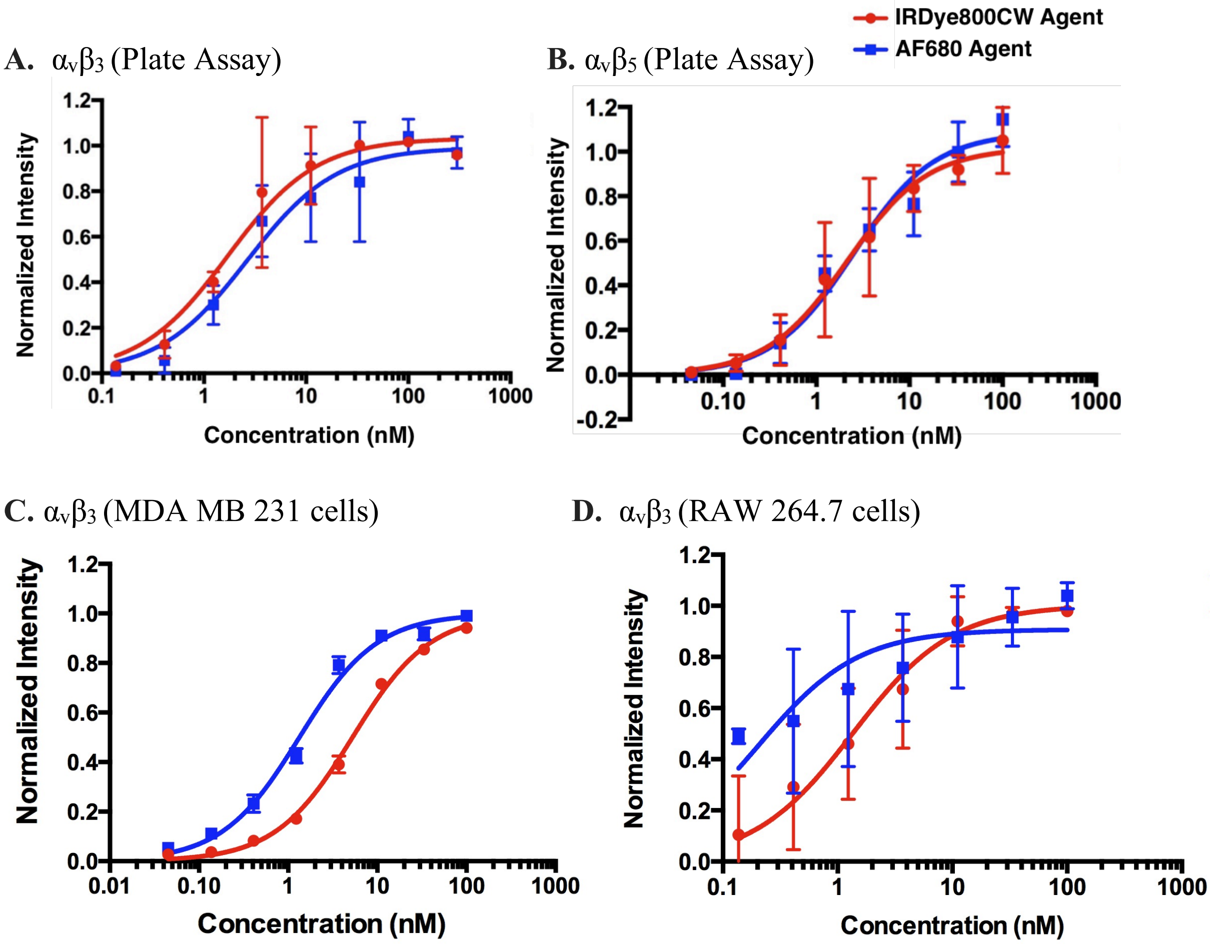

Figure S1. Binding affinity of the IRDye800CW and AF680 agent to integrin of the form (A) human $\alpha_v\beta_3$ on a plate, (B) human $\alpha_v\beta_5$ on a plate, (C) human $\alpha_v\beta_3$ on MDA MB 231 and (D) mouse $\alpha_v\beta_3$ on RAW 264.7 cells.

Type of Assay	Integrin	Species	IRDye800CW Agent	AF680 Agent
Plate	$\alpha_v\beta_3$	Human	1.7 ± 0.3 nM	2.7 ± 0.6 nM
	$\alpha_v\beta_5$	Human	1.5 ± 0.4 nM	2.1 ± 0.4 nM
Cell based	$\alpha_v\beta_3$ (HEK293 Transfected)[1]	Human	5.1 ± 2.5 nM	0.3 ± 0.1 nM
	$\alpha_v\beta_3$ (MDA MB 231)	Human	5.1 ± 0.7 nM	1.3 ± 0.2 nM
	$\alpha_v\beta_3$ (RAW 264.7)	Mouse	0.7 ± 0.3 nM	1.1 ± 1.7 nM

The measured dissociation constants were generally in the low single-digit nanomolar with some variance depending on the assay type (recombinant proteins measured on plates versus cells) and species (human versus mouse).

Figure S2. Healthy Mice Dosed Subcutaneously (SC)

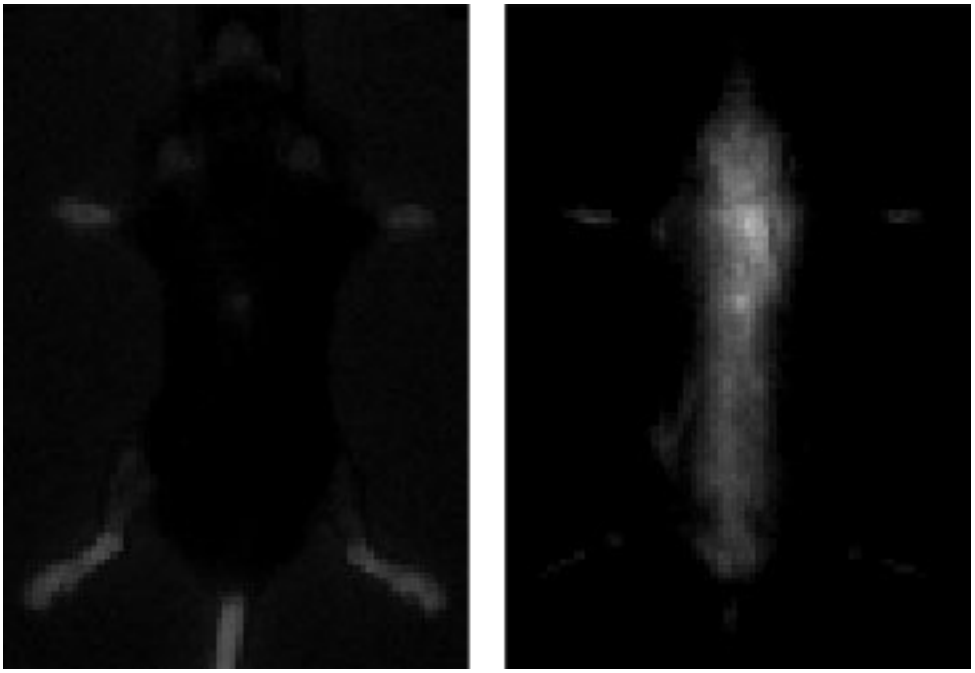

Figure S2. IVIS images of healthy mice at 48 hours after co-administration of the (left) IRDye800CW and (right) AF680 agents. Quantification of the signal from the joints is shown in Fig 2.

Figure S3. Plasma Clearance Curves

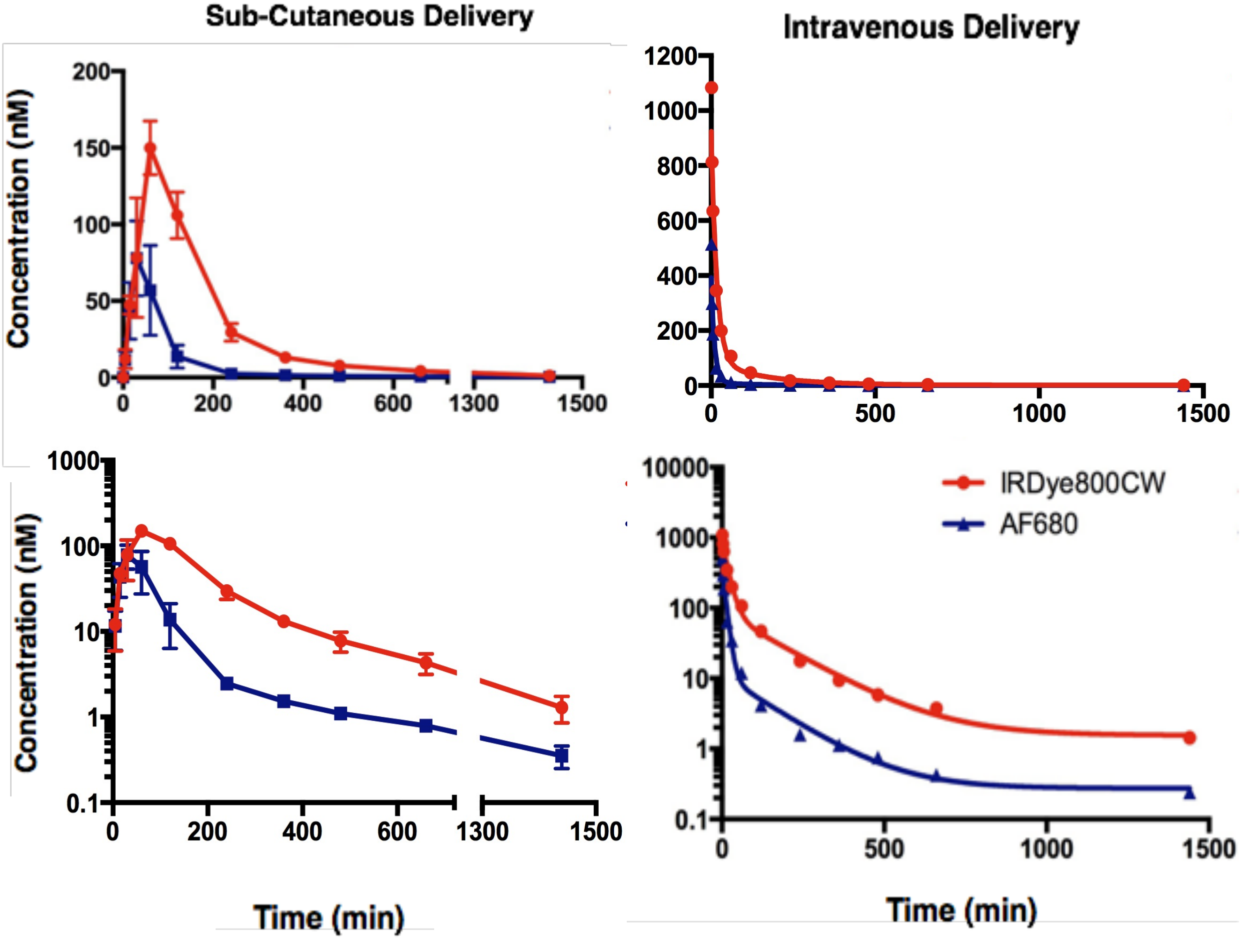

Figure S3. Plasma clearance curves (linear, top and semi-log bottom) for sub-cutaneous and intravenous delivery for the IRDye800CW and AF680 agents at a dose of 1.5 nmoles.

For IV Delivery:

Co (IRDye800CW Agent) = 1271nM
Co (AF680 Agent) = 693nM

Percent Fast (IRDye) = 90.69
Percent Fast (AF680) = 97.20

Kfast (IRDye) = 0.058 /min
Kfast (AF680) = 0.095/ min

Kslow (IRDye) = 0.0062 /min
Kslow (AF680) = 0.007 /min

Figure S4. Healthy Mice Dosed Orally (PO)

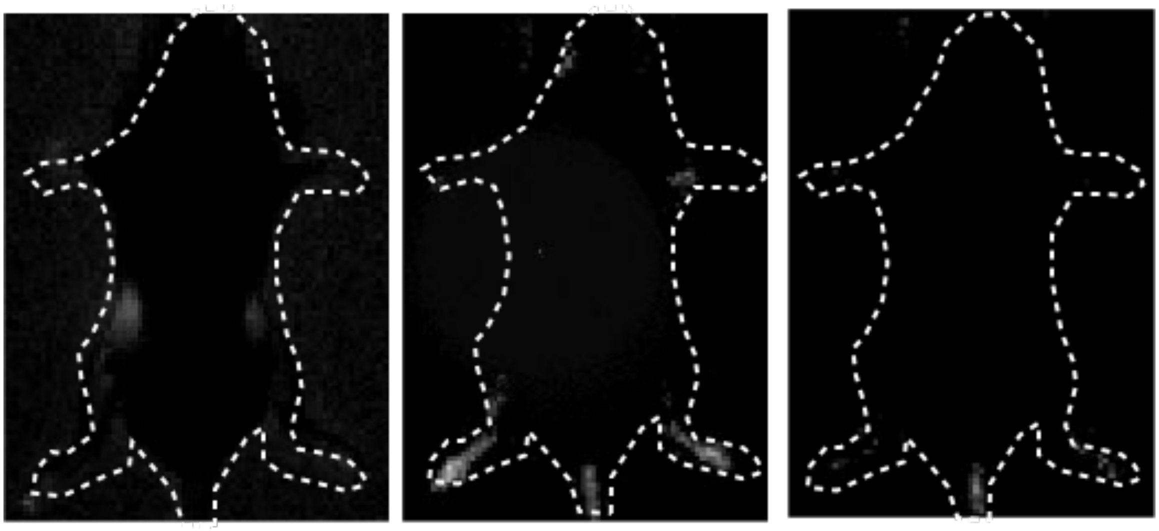

Figure S4. IVIS images of healthy mice at (left) 6, (middle) 24 and (right) 48 hours after oral administration of the IRDye800CW agent. Quantification of the signal from the joints is shown in Fig 3.

Figure S5. Autofluorescence Controls for Confocal Images

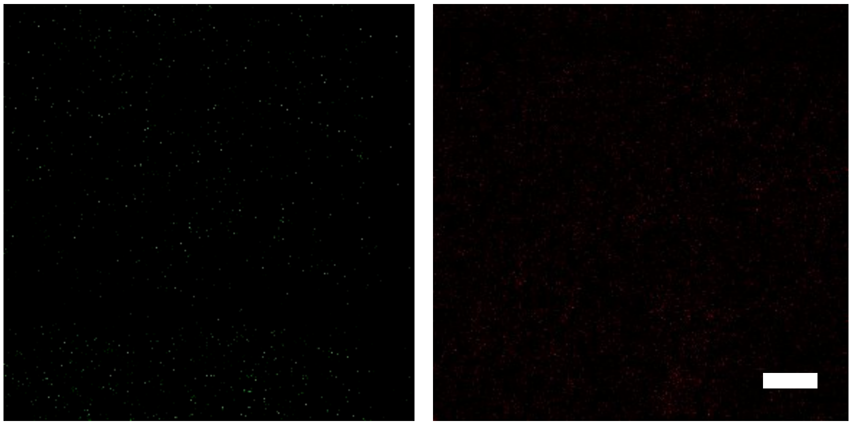

Figure S5. Autofluorescence controls for the (left) IRDye800CW agent signal and (right) macrophage staining.

The autofluorescence samples were obtained from joints of healthy mice that were not administered the imaging agent.

Figure S6. CAD Model of Synovial Membrane

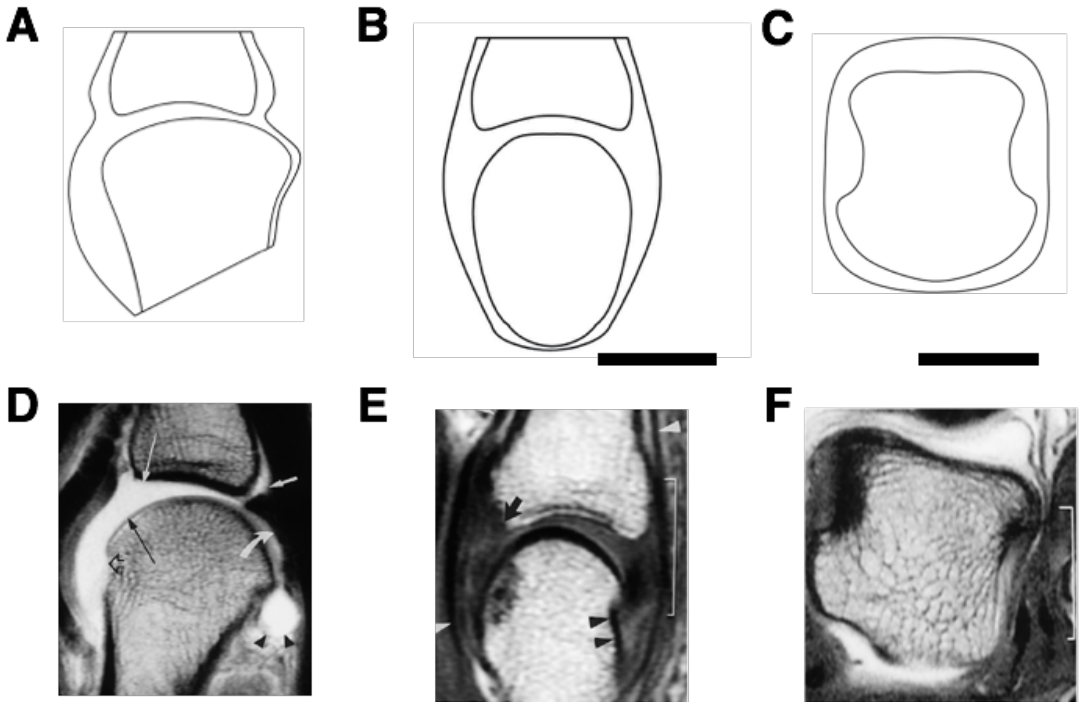

Figure S6. Model of the synovial membrane based on MR images from Theumann et al[2]. The CAD model (A-C) shown next to MRI images (D-F) showing sagittal (left), coronal (middle), and transverse (right) images of the synovial space. Scale bar = 10 mm

Figure S7. Optical Phantoms

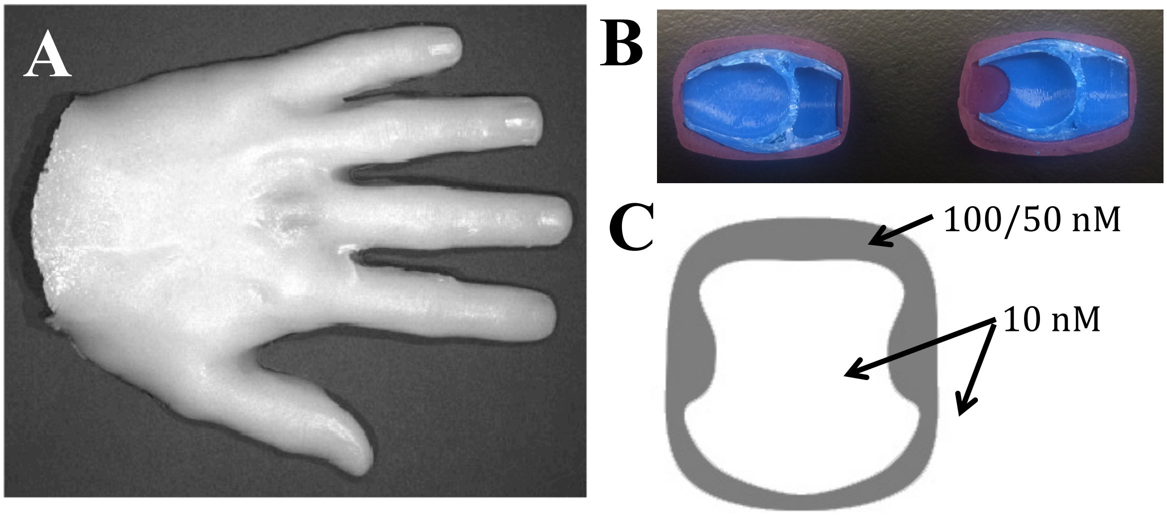

Figure S7. (A) A silicone mold of a human hand was used to generate the optical phantom. (B) The CAD model of the synovium was used to 3D print a cast (blue) to generate a mold (purple) for the joint. (C) Cross-section of optical phantom, with the darker region being the joint. The grey region had either 100 or 50 nM of the dye and the healthy tissue in white had 10 nM of the dye.

Figure S8. Concentration Ratio Scale Up

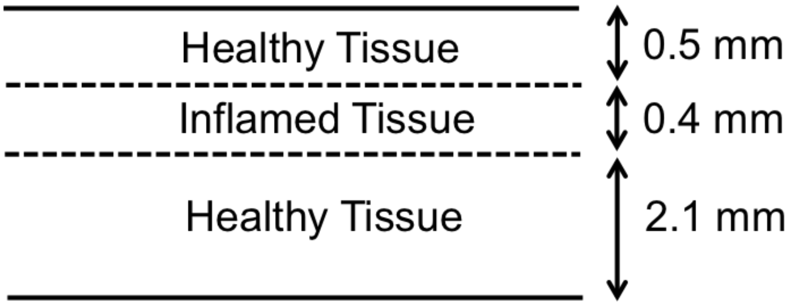

Figure S8. Schematic of inflamed paw with epifluorescence imaging (excitation and emission occurring from the top). A healthy paw would have an identical schematic without the inflamed tissue.

Average healthy paws: 2.6mm
Average extent of inflammation: 0.4mm
Imaging intensity ratio between inflamed paws and healthy paws (TBR) = 4

Two approaches were used to scale the imaging signal intensity to the concentration ratio between inflamed and healthy paws: a simple linear scaling shown below and a diffuse light approximation of fluorescence intensity[3]. Both results gave similar answers, which was anticipated given the small thickness of the mouse paw relative to NIR light penetration. For the linear scaling, let the concentration in the inflamed tissue be X nM and in the healthy tissue be Y nM.

$$TBR = \frac{Concentration\ in\ Inflamed\ Paw}{Concentration\ in\ Healthy\ Paw}$$

$$TBR = \frac{0.5\ (Y) + 0.4\ (X) + 2.1\ (Y)}{2.6\ (Y)} = 4$$

Solving the equation, we get $\frac{X}{Y} = 19.5$

The concentration ratio of inflamed to healthy tissue is 19.5

Based on a z-statistic and the diffuse light simulations, a 5.5-fold concentration ratio between the inflamed region and surrounding tissue is needed for positive identification above the variability in background signal. If a similar targeting efficiency occurs in humans, this technique will translate efficiently to clinical depths.

Figure S9. IRDye800CW Agent Absorption in Mice

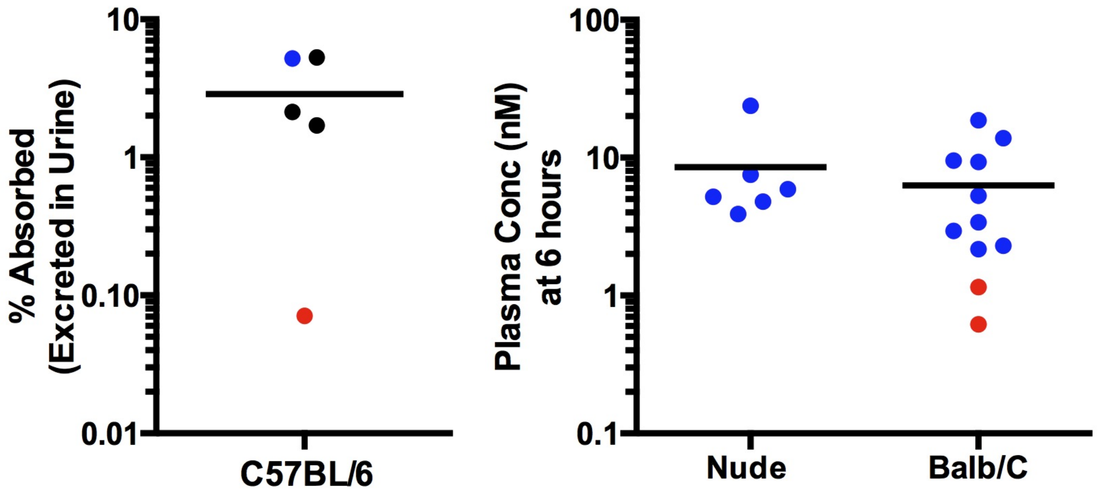

Figure S9. Absorption of the imaging agent across different strains of mice with mice having low absorption shown in red. Black – 1mg/kg dose. Blue – 5mg/kg dose. The low absorption in C57BL/6 was seen at a 1mg/kg dose whereas the low absorption in Nudes and Balb/C was seen at a 5mg/kg dose.

Low absorption was seen in 14% of mice across all species and dosages and was seen in 18% of of Balb/C mice with a dose of 5mg/kg. As mentioned in the discussion section, mice with low absorption could either be administered an increased oral dose or subcutaneous dose of the imaging agent.

Table S1. Parameters used in COMSOL Model

Parameter	Value	Reference	Description
μ_a	$20\ m^{-1}$	[4,5]	Absorption coefficient
D	2.732×10^{-4} m		Diffusion coefficient
S	$4.1\ W/m^2$	[3]	Excitation light source
C_{back}	$2.5 \times 10^{-5}\ mol/m^3$	[6]	25% of the plasma concentration
$\eta_{IRDye\text{-}800CW}$	0.1	[7]	IRDye-800CW quantum efficiency
$\varepsilon_{IRDye\text{-}800\ CW}$	$24{,}000\ m^3/mol/m$		IRDye-800CW extinction coefficient
$\varepsilon_{emission}$	24%	[8]	Emission light percentage absorption
C_r	2.94	[9]	Boundary condition constant
μ_s	$12000\ m^{-1}$	[4,5]	Tissue scattering coefficient
g	0.9	[4]	Scattering anisotropy
μ_s'	$1200\ m^{-1}$		Reduced scattering coefficient
R_{eff}	0.493	[10]	Effective reflection constant

Table S2. Equations

Parameter	Equation	Description
μ_s'	$\mu_s' = \mu_s(1 - g)$	Reduced scattering coefficient
D	$D = 1/3(\mu_a - \mu_s')$	Diffusion coefficient
$\mu_{a\_ex\_back}$	$\mu_{a\_ex\_back} = \mu_a + \varepsilon_{IRDye}C_{back}$	Effective absorption coefficient (background, excitation)
$\mu_{a\_ex\_mem}$	$\mu_{a\_ex\_mem} = \mu_a + \varepsilon_{IRDye}C_{mem}$	Effective absorption coefficient (membrane, excitation)
$\mu_{a\_em\_back}$	$\mu_{a\_em\_back} = 0.5(\mu_a + \varepsilon_{IRDye}C_{back})$	Effective absorption coefficient (background, emission)
$\mu_{a\_em\_mem}$	$\mu_{a\_em\_mem} = 0.5(\mu_a + \varepsilon_{IRDye}C_{mem})$	Effective absorption coefficient (membrane, emission)
C_r	$C_r = (1 + R_{eff})/(1-R_{eff})$	Boundary condition constant

Planar light source diffusion equation and corresponding Helmholtz equation.

The term *f* of on the right side of Helmholtz equation is the source term.

$$\nabla \cdot (-c\nabla u) + au = f$$

To simulate a planar source with a value of f = 4.1 W/m^3 in COMSOL, there are direct options for three-dimensional source.

For planar source, there is no explicit representation for *f*. So choosing Flux/Source, where

$$-\mathbf{n} \cdot (-c\nabla u) = g - qu$$

Rearrange the Helmholtz Equation,

$$\nabla \cdot (-c\nabla u) = f - au$$

So, assign $f = 4.1 = g$ and q = 0 for the boundary conditions.

Boundary conditions equation and corresponding Helmholtz equation.

From COMSOL Helmholtz equation

$$-\hat{n}(-D \cdot \nabla u) + qu = 0$$

Rearrange the equation

$$\mathrm{D} \cdot \nabla u + qu = g$$

$$\frac{\partial \mathrm{u}}{\partial \mathrm{r}} + qu = g$$

Rearrange the diffusion equation

$$-2Cr \cdot D \cdot \frac{\partial \Phi}{\partial r} + \Phi = 0$$

Compare the two equations, the corresponding coefficients are

$$\mathrm{g} = 0$$

$$\mathrm{q} = \frac{1}{2Cr}$$

Limitations of Model

There are several limitations to the COMSOL model. First, the 3D synovial space and hand are only approximations of the complex tissue structures present within the hand, which may impact light propagation. When drawing the joint in Solidworks, the exact 2D sketches of the joint were extracted from the three anatomical planes of its MR images (sagittal, coronal and transverse view). Then the 3D model was constructed by the lofting feature in Solidworks, using the three sketches of top, middle and bottom planes of the transverse view as bases, and sketches on sagittal and coronal planes as guidelines. Thus, although the model has the same anatomical cross-section views as the MR images, the overall 3D shape is an approximation based on the available MR images. Second, the optical phantom is also an experimental approximation of the behavior of light in living tissue. There are no internal boundaries in the optical phantom, and the absorption and scattering are homogeneous average values for tissue. Third, the variability in background signal intensity was estimated based on animal data and limited clinical data with ICG and could vary from the estimated value.

Table S3. TBR and CNR for varying Concentration Ratios

IRDye-800CW	TBR	Target to Background Concentration Ratio 10:1	5:1	2:1	1:1
Joint Swelling Percentage	0%	3.1 ± 0.1	1.92 ± 0.09	1.23 ± 0.06	1.04 ± 0.05
	5%	3.2 ± 0.1	1.98 ± 0.09	1.25 ± 0.06	1.04 ± 0.05
	10%	3.3 ± 0.1	2.01 ± 0.09	1.26 ± 0.06	1.04 ± 0.05
	20%	3.4 ± 0.2	2.09 ± 0.09	1.29 ± 0.06	1.04 ± 0.05

IRDye-800CW	CNR	Target to Background Concentration Ratio 10:1	5:1	2:1	1:1
Joint Swelling Percentage	0%	3.3 ± 0.2	1.5 ± 0.1	0.37 ± 0.09	0.07 ± 0.08
	5%	3.4 ± 0.2	1.5 ± 0.1	0.39 ± 0.09	0.07 ± 0.08
	10%	3.5 ± 0.2	1.6 ± 0.1	0.41 ± 0.09	0.07 ± 0.07
	20%	3.8 ± 0.2	1.7 ± 0.1	0.44 ± 0.09	0.07 ± 0.07

References

1 Bhatnagar, S. *et al.* Oral Administration and Detection of a Near-Infrared Molecular Imaging Agent in an Orthotopic Mouse Model for Breast Cancer Screening. *Molecular pharmaceutics*, doi:10.1021/acs.molpharmaceut.7b00994 (2018).

2 Theumann, N. H., Pfirrmann, C. W., Drape, J. L., Trudell, D. J. & Resnick, D. MR imaging of the metacarpophalangeal joints of the fingers: part I. Conventional MR imaging and MR arthrographic findings in cadavers. *Radiology* **222**, 437-445, doi:10.1148/radiol.2222010181 (2002).

3 Thurber, G. M., Figueiredo, J. L. & Weissleder, R. Detection Limits of Intraoperative Near Infrared Imaging for Tumor Resection. *J. Surg. Oncol.* **102**, 758-764, doi:10.1002/jso.21735 (2010).

4 Wang L., W. H. *Biomedical Optics: Principles and Imaging*. (Wiley, 2007).

5 Weissleder, R. A clearer vision for in vivo imaging. *Nat Biotech* **19**, 316-317 (2001).

6 Thurber, G. M., Zajic, S. C. & Wittrup, K. D. Theoretic criteria for antibody penetration into solid tumors and micrometastases. *Journal of nuclear medicine : official publication, Society of Nuclear Medicine* **48**, 995-999 (2007).

7 Adams, K. E. *et al.* 9 (SPIE).

8 L'Huillier, J.-P. & Vaudelle, F. Improved localization of hidden fluorescent objects in highly scattering slab media based on a two-way transmittance determination. *Opt. Express* **14**, 12915-12929, doi:10.1364/OE.14.012915 (2006).

9 Farrell, T. J., Patterson, M. S. & Wilson, B. A diffusion theory model of spatially resolved, steady-state diffuse reflectance for the noninvasive determination of tissue optical properties in vivo. *Medical physics* **19**, 879-888, doi:10.1118/1.596777 (1992).

10 Schmidt, M. M. & Wittrup, K. D. A modeling analysis of the effects of molecular size and binding affinity on tumor targeting. *Molecular cancer therapeutics* **8**, 2861-2871, doi:10.1158/1535-7163.mct-09-0195 (2009).
